# Optimization of flow cytometric detection and cell sorting of transgenic *Plasmodium* parasites using interchangeable optical filters

**DOI:** 10.1186/1475-2875-11-312

**Published:** 2012-09-05

**Authors:** Ivan A Vorobjev, Kathrin Buchholz, Prashant Prabhat, Kenneth Ketman, Elizabeth S Egan, Matthias Marti, Manoj T Duraisingh, Natasha S Barteneva

**Affiliations:** 1 Immune Disease Institute and Program in Cellular and Molecular Medicine, Children’s Hospital, D-239, 200 Longwood Avenue, 02115, Boston, MA, USA; 2 A.N. Belozersky Institute for Physico-Chemical Biology and Department of Biology, M.V. Lomonosov Moscow State University, Moscow, Russia; 3 Department of Immunology and Infectious Diseases, Harvard School of Public Health, Boston, MA, USA; 4 Semrock Inc, Rochester, NY, USA

**Keywords:** Malaria, *Plasmodium*, Optical filter, Fluorescent proteins, Cell sorting, Rare cells

## Abstract

**Background:**

Malaria remains a major cause of morbidity and mortality worldwide. Flow cytometry-based assays that take advantage of fluorescent protein (FP)-expressing malaria parasites have proven to be valuable tools for quantification and sorting of specific subpopulations of parasite-infected red blood cells. However, identification of rare subpopulations of parasites using green fluorescent protein (GFP) labelling is complicated by autofluorescence (AF) of red blood cells and low signal from transgenic parasites. It has been suggested that cell sorting yield could be improved by using filters that precisely match the emission spectrum of GFP.

**Methods:**

Detection of transgenic *Plasmodium falciparum* parasites expressing either tdTomato or GFP was performed using a flow cytometer with interchangeable optical filters. Parasitaemia was evaluated using different optical filters and, after optimization of optics, the GFP-expressing parasites were sorted and analysed by microscopy after cytospin preparation and by imaging cytometry.

**Results:**

A new approach to evaluate filter performance in flow cytometry using two-dimensional dot blot was developed. By selecting optical filters with narrow bandpass (BP) and maximum position of filter emission close to GFP maximum emission in the FL1 channel (510/20, 512/20 and 517/20; dichroics 502LP and 466LP), AF was markedly decreased and signal-background improve dramatically. Sorting of GFP-expressing parasite populations in infected red blood cells at 90 or 95% purity with these filters resulted in 50-150% increased yield when compared to the standard filter set-up. The purity of the sorted population was confirmed using imaging cytometry and microscopy of cytospin preparations of sorted red blood cells infected with transgenic malaria parasites.

**Discussion:**

Filter optimization is particularly important for applications where the FP signal and percentage of positive events are relatively low, such as analysis of parasite-infected samples with in the intention of gene-expression profiling and analysis. The approach outlined here results in substantially improved yield of GFP-expressing parasites, and requires decreased sorting time in comparison to standard methods. It is anticipated that this protocol will be useful for a wide range of applications involving rare events.

## Background

*Plasmodium falciparum* remains one of the world’s most devastating infections with official estimates of mortality from malaria ranging from 800,000 deaths to 1.2 million deaths per year [1,2]. The development of transgenic *Plasmodium* lines expressing green or red fluorescent proteins has enhanced the study of the parasite’s different life cycle stages through cell biological and, more recently, flow cytometry-based methods [3-6]. Furthermore, such transgenic lines represent excellent tools for growth-inhibition assays and parasite characterization [7-9]. However, red blood cells (RBC) exhibit strong autofluorescence (AF) at 620 nm due to the presence of intermediate products of haem metabolism (protoporphyrin) [10], and in the range of 500–540 nm, which overlaps the excitation/emission spectrum of GFP. Therefore, the major aim of this study was to optimize filter sets in order to improve the detection of GFP-expressing transgenic parasites. In addition, another hurdle must be overcome when sorting rare populations, which is essential for examination of the malaria life cycle *in vivo*: how to obtain sufficient cell numbers while ensuring high purity of the sorted cell population. Here, data are presented demonstrating that both of these issues can be overcome easily and cost-effectively. Based on recent advances in flow cytometer optical filter interchangeability, stages of the *Plasmodium falciparum* transmission cycle expressing GFP or tdTomato, respectively, were effectively detected and isolated. Optimization of optical filters allowed for the detection of 50-150% more GFP-expressing parasites as compared to use of filters supplied with the flow cytometer, and sorted populations were >90% pure. This can advance *Plasmodium* gametocyte research, since the challenge in isolating these parasite stages often lies in either low overall number of gametocytes in certain parasite lines or in contamination of rare desired events (e.g. very young gametocytes or mature gametocytes out of a mixed population) with unwanted parasite stages (e.g. asexual stages). Based on the approaches described here, investigators will be able to rapidly evaluate optical filter efficiency using cell number and desired purity for a given level of statistical significance.

## Methods

### *In vitro* culture of *Plasmodium falciparum* parasites and preparation of cells prior to flow cytometry analysis

Three *P. falciparum* lines were mainly used in this study: a non-fluorescent gametocyte-producing clone termed P2G12 derived from the reference strain 3D7 (P2G12 WT, [9]); and two transgenic lines derived from this clone which express GFP (164/GFP, [9]), and tandem dimer tomato fluorescent protein (164/tdT, [11]), respectively, from the gametocyte-specific promoter of the gene PF10_0164. Furthermore, to confirm findings with other GFP-expressing parasite lines, this study used the *P. falciparum* 3D7/pMAL13P1.130-GFP line, harbouring a plasmid that encodes GFP under the control of a schizont-stage promoter (schizont/GFP, gift from T. Gilberger) [12], and a *P. falciparum* line that constitutively expresses GFP from an integrated construct [13]. All parasites were cultured *in vitro* as described elsewhere [14]. Briefly, parasites were maintained in fresh type 0+ human erythrocytes (Research Blood Components, Boston, MA, USA) in complete medium containing 1% AlbuMAX II (Life Technologies, Grand Island, NY, USA), 0.5 ml of gentamycin, 5.94 g HEPES, 2.01 g of sodium bicarbonate, 0.05 g hypoxanthine, and 10.44 g Roswell Park Memorial Institute-1640 per litre (pH 6.74). The Harvard University Ethics Committee approved use of erythrocytes from human donors. Parasite cultures were kept at 37°C in gassed chambers at 5% CO_2_ and 1% O_2_. Parasite preparation prior to flow cytometry analysis varied for the different lines. P2G12 WT, 164/GFP and 164/tdT were cultured in T75 flasks without a specific gametocyte-induction protocol to obtain baseline gametocyte production in asexually replicating cultures. Prior to flow cytometry analysis, all cultures were subjected to Percoll gradient enrichment to obtain asexual late blood-stage parasites and mature gametocytes. Enriched parasites were resuspended in RPMI-1640 medium (no phenol red) and 20% human serum. For direct comparison of the effect of fixation on flow cytometric detection, all three parasite lines were cultured alongside one another and split after purification, after which parasites were left unfixed or partially fixed using 0.1% paraformaldehyde (PFA) in PBS/0.1% BSA. The schizont/GFP transgenic parasites were cultured in human red blood cells. These parasites were synchronized using standard protocols; the resulting mature schizont stages were fixed as described above and subsequently used for flow cytometry analysis. Parasites that constitutively express GFP were sorbitol synchronized to obtain ring stage parasites and were also fixed as described above.

### Flow cytometry sorting and analysis

Two FACSAria II flow cytometers (BD Biosciences, San Jose, CA, USA) equipped with a combination of 407 nm, 488 nm, 561 nm, 640 nm, or 355 nm, 407 nm, 488 nm, 593 nm, and 640 nm lasers were used for analysis and cell sorting. Sorted cells were deflected into Falcon tubes containing PBS + 10% fetal calf serum (FCS). All experimental procedures with non-fixed cells were performed according to biosafety BL2^+^ level practice. To avoid the sorting of cell doublets or cell aggregates, single cells were sequentially selected on FSC-H/FSC-W and SSC-H/SSC-W dot plots. Sorting parameters included: i) sheath pressure set at 45 psi; and ii) use of an 85 μm nozzle tip. To acquire the GFP signal the following bandpass (BP) filters were compared: 514/30, 510/21, a set of two different 530/30 filters purchased together with the cytometers, and 510/20, 512/20, 517/20, 529/24 filters provided by from Semrock Inc (Rochester, NY, USA). To acquire the tdTomato signal, two BP filters were compared: a 576/26 optical filter purchased with the instruments and a 585/29 filter provided by Semrock Inc (spectral information on the filters is summarized in Additional file 1). As a dichroic filter a 502 longpass filter (502LP) was used (results of some experiments comparing 502LP vs 466LP fil-ter (LM01-466Di02 from Semrock Inc, which at 11 degree angle of incidence has its edge at 502 nm are summarized in Additional file 2).

The autofluorescent (AF) intensity ratio was calculated using FL1/FL2 channels for the acquisition of negative ER (non-expressing FP). The FL1 channel (used for GFP acquisition) approximately corresponds to the peak of fluorescence of oxidized flavins (520–540 nm) [15,16].

### Filter transmission measurement

All the filter spectra were measured using a custom-built spectrophotometer at Semrock, Inc. Additional details on the instrumentation and the measurement methods are provided in a separate white paper [17]. This instrument utilizes a double monochromator with a cooled, UV-enhanced CMOS camera in order to perform sensitive spectral measurements such as transmission through optical filters with very steep edges and high blocking.

### Imagestream 100 analysis of sorted infected erythrocytes

After FACS sorting, sorted parasites were transferred into 50 μl tubes and subjected to imaging cytometry analysis with the Imagestream 100 (Amnis Inc, Seattle, USA). Imagery and cytometric analysis was performed using IDEAS software from Amnis Inc.

### Microscopy of cytospin preparation

Cytospin slide centrifugation was used to concentrate 100 μl of sorted parasite sample for Giemsa staining. Each sample was pipetted into a plastic chamber, placed in a cytospin slide centrifuge (Cytospin 2, Shandon Southern Instruments, Inc, Sewickley, PA, USA) and centrifuged for 5 min at a set speed of 100 RPM. Parasites were deposited in a 7 mm-circular area on the slide, air dried and then Giemsa-stained for 15 min. Cytospin smears were subsequently investigated under a light microscope (Axiostar plus, Zeiss Inc, Thornwood, NY, USA) and photomicrographs were taken.

### Statistical analysis

Files of 10^5^-10^6^ events were acquired by flow cytometer. The data were analysed by Diva 6.1 (BD Biosciences, San Jose, CA, USA) and FlowJo software (Treestar, Ashland, OR, USA). Student’s t-test was used to compare data from cytometry experiments. The level of significance was set at P < 0.05. All data are representative of three independent experiments.

## Results

**Figure 1:**
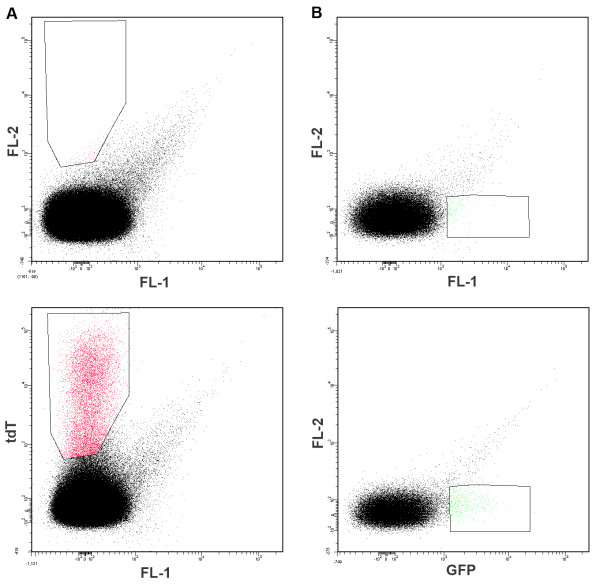
**Representative dot blots of red blood cells infected with either non-fluorescent *P. falciparum *(P2G12-WT) (A and B) or with *P. falciparum*expressing tdT (164/tdT) (A) or GFP (164/GFP) (B), respectively (BP filters 575/25 and 530/30, accordingly).** The upper panel represents the non-fluorescent control parasites and the lower panel shows the fluorescent parasites populations.

**Figure 2:**
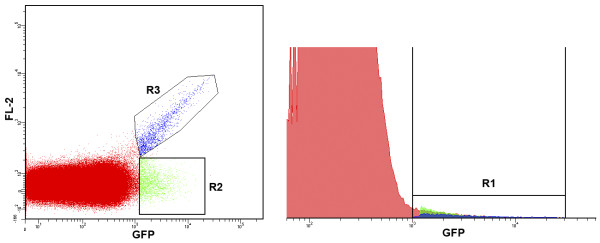
True positive events (R2) of the GFP-expressing parasite line 164/GFP cannot be properly selected using histogram analysis, where R2 and R3 (high autofluorescence) (left side) merge into R1 (BP filter 530/30, right side).

When attempting to detect rare events two relatively straightforward approaches can be taken to increase sensitivity: increasing the positive signal or reduction of AF. As a first step to achieve improved sensitivity when detecting malaria parasites in infected red blood cells by flow cytometry, an attempt to reduce AF was undertaken. When sexual parasites harboring a vector expressing tdTomato (emission max = 581 nm) [18] were compared to non-fluorescent wild-type parasites, good separation of tdTomato^+^ and tdTomato^-^ events was obtained (FL2 channel; emission filter 576/26) (Figure 1A). The same results were obtained using a 585/29 emission filter (data not shown). However, when the standard emission filter supplied with the instrument (FL1 channel; emission filter 530/30) was used for a similar analysis of sexual parasites harbouring a vector expressing GFP, the separation between positive and negative events was compromised due to high levels of AF in the FL1 channel **(**Figure 1B**)**. This background signal appears to be critical impediment for successful sorting of the GFP^+^ parasite population. The same results were found when using GFP-expressing ring- and schizont-stage parasites (data not shown). As shown in Figure 2, when events from the GFP expressing sample are presented in two-dimensional dot blot and histogram formats and compared, it is clear that the histogram format is not useful for proper gating of GFP^+^-events. On the histogram “diagonal” AF (R3 on Figure 2, left) will merge with R2 (on Figure 2, left) to form part of what will be treated as the GFP^+^-subpopulation (R1 on Figure 2, right). 

**Figure 3:**
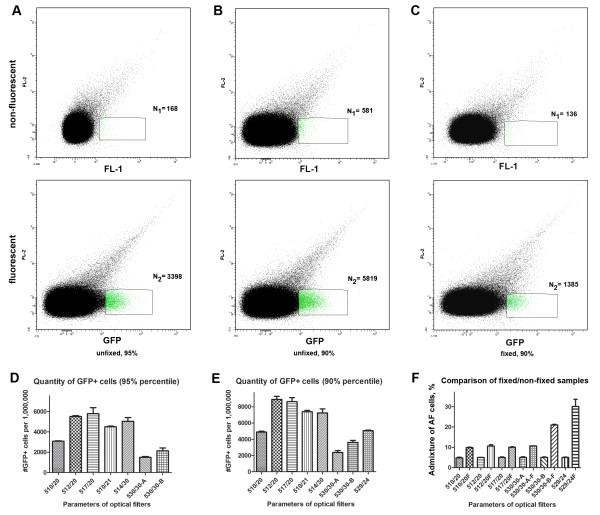
**A, B, C Gating strategy of dot blots of non-fluorescent (P2G12-WT) and transgenic GFP**^**+ **^***P. falciparum*-infected red blood cells (line 164/GFP) for 95% (A) and 90% (B) purity, and 90% (C) purity for a fixed sample (line 3D7/pMAL13P1.130-GFP).** In A-C the upper panel represents the non-fluorescent parasites and the lower panel the corresponding fluorescent parasites. **D.** Amount of transgenic GFP-expressing *P. falciparum* gametocytes (line 164/GFP) acquired on a FACSAria cytometer using different filters: 510/20; 512/20, 517/20; 510/21; 514/30; 530/30-A; 530/30B (calculated for 95% purity per 1,000,000 acquired events). Data from three separate experiments were analyzed. 530/30-A and 530/30-B represent two optical filters originally supplied with FACSAria cytometers for the FL1 channel. **E.** Same as Figure 3D, with 90% purity. **F.** Percent of autofluorescent events admixture acquired on a FACSAria sorter using the above-mentioned filters and the additional filter 529/24. Red blood cells infected with GFP-expressing parasites (line 3D7/pMAL13P1.130-GFP) before or after 0.1% PFA fixation were used. For each filter two values are given, in which the filter name labeled with “F” represents the value for the fixed sample.

To estimate the FL1 region for the detection of positive events, signals obtained from unfixed GFP-expressing parasites and from non-fluorescent parasite-infected RBC were analysed using a two-dimensional dot plot (GFP (FL1) channel vs FL2 channel). The yield of positive events that could be obtained for GFP^+^ specimens was calculated as follows: on the dot plot of the fluorescent parasite sample the region containing the major part of the positive population was taken and placed in a way to include a minimal amount of events in the control (non-fluorescent) specimen (Figure 3A). First, the number of events that could be detected with purity of not less than 95% was calculated. To achieve this, the region was moved until the ratio between the number of events detected in the region on the non-fluorescent cell plot versus the region on the fluorescent cell plot reached 1:20. A representative example of this gating is shown in Figure 3A. Next, events in this defined gate were counted using the different optical filters (Figure 3D**)**. These included the standard 530/30 FL1 filters supplied with two different cytometers (designated 530/30-A and 530/30-B, respectively), and five other bandpass (BP) filters designated as 510/20, 512/20, 517/20, 510/21, and 514/30. The transmission spectra of the filters tested are given in Additional file 1A. The emission spectrum of GFP [19] with the overlay of the spectra of several filters (512/20; 517/20; 530/30-A and 530/30-B) can be found in Additional file 1B. 

Using the 512/20 and 517/20 optical filters yielded >2-fold more events in the defined gate than the stand-ard 530/30 filters purchased with the computers (Figure 3D). Since the overall yield of GFP^+^ cells was quite low at >95% purity (<1%), regions were changed for all filters to achieve 90% purity of the selected subpopulation in order to enhance yield **(**Figure 3B, E**)**. Once again, use of the 512/20 and 517/20 filters resulted in the best yield of GFP^+^ cells. Finally, to determine the effect of fixation on sorting purity and yield, non-fluorescent and GFP-expressing parasites (3D7/pMAL13P1.130-GFP) were fixed and sorted with gating to 90% purity using a FACSAria II SORP and optimized GFP-filter. The fixation led to a 2–2.5-fold increase in the admixture of autofluorescent cells with the optimal filters (Figure 3F). However, an even bigger admixture of autofluorescent cells was observed for non-optimal filters (530/3-A, 530/30B and 529/24), ranging from 4.1 to 5-fold increase (Figure 3F). Thus to achieve 90% purity of a fixed cell population, sorting yield will be decreased even using the best filters.

The maximal yield achieved with filters 512/20 and 517/20 for GFP-expressing parasites was close to that obtained with tdT-expressing parasites (8,620 and 8,480 per 1×10^6^ erythrocytes), a system that is more ideal since tdT has an emission peak in a region where autofluorescence from RBC is low. A BP-filter 510/20 centred at the GFP emission peak (509 nm) resulted in a poorer yield than the 512/20 and 517/20 filters (when used with 502LP dichroic). Thus, based on these findings, separation filters with a centre of emission bandwidth slightly shifted (2–3 nm) to the right of the GFP emission peak have to be used for maximum yield of GFP-expressing parasite-infected cells. However, BP-filter 510/20 provided the best yield when used with optimized dichroics filter 466LP (Additional file 2).

**Figure 4:**
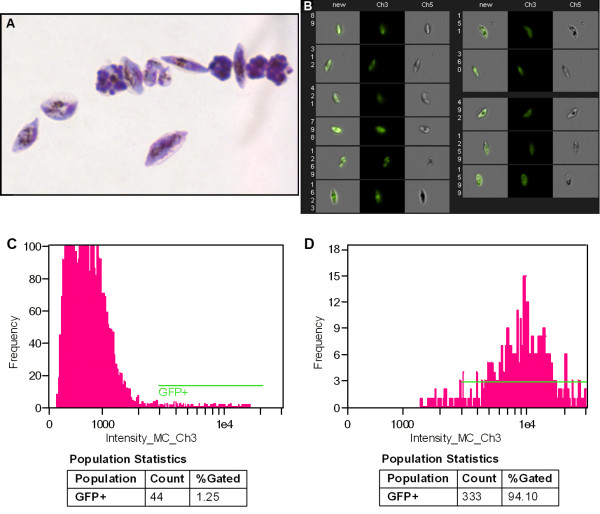
**A, B, C, D Image analysis of sorted red blood cells infected with GFP-expressing transgenic parasites from line 164/GFP. A.** Microscopic image of sorted red blood cells infected with GFP-expressing transgenic parasites from line 164/GFP (cytospin, Giemsa staining, Objective ×100/1.3 Oil). **B.** Gallery of images of sorted red blood cells infected with GFP-expressing parasites (line 164/GFP) acquired with Imagestream 100 imaging cytometer. Left channel: bright field, middle channel: fluorescent channel 3 (GFP-channel), right channel: merged bright field and green channels. **C.** Histogram of unsorted red blood cells infected with GFP-expressing transgenic parasites from line 164/GFP. **D.** Histogram of sorted red blood cells infected with GFP-expressing transgenic parasites (164/GFP).

Although erythrocytes are fragile cells, successful FACS sorting of iRBCs can be achieved by lowering pressure and using additional precautions [7,18,20]. The purity and yield of the sorted populations of iRBCs infected with GFP-expressing parasites was further analysed under the microscope after cytospin enrichment and Giemsa staining (Figure 4A) and by Imagestream 100 (Figure 4B-D, Additional file 3). Imaging cytometry analysis verified that the GFP^+^ gametocytes represent approximately 93-95% of the sorted population (Figure 4D). The percentage of GFP^+^ events of the unsorted iRBCs mixture shown on the imaging cytometry histogram (Figure 4C) is higher than the yield: this can be explained by the inclusion of some autofluorescent events. The yield of sorted GFP^+^ gametocytes differed between samples sorted with a 530/30 filter and a 512/20 filter differs by 2.5 to 3 fold as shown in Figure 3. Sorting with the 512/20 BP filter allowed for the collection of 150-200% more GFP^+^ gametocytes after additional optimization of the dichroic filter was performed (466LP > 502LP-Additional file 2). Furthermore, the improved filter set for GFP^+^ event detection gave consistent results in several independent biological replicates (Table 1) with purity in the high 90%. 

**Table 1:** **Cytometric analysis of *Plasmodium falciparum *GFP**^**+**^**gametocytes (line 164/GFP) before and after sorting**

**Cell population**	**% GFP**^**+**^**cells-Exp1**	**% GFP**^**+**^**cells-Exp2**	**% GFP**^**+**^**cells-Exp3**
Before sorting	0.05%	0.06%	0.03%
After sorting	95.1%	94.7%	93.1%

Gametocytes were collected in an equal volume of PBS supplemented with 1% BSA, HEPES and L-glutamine, subsequently subjected to imaging cytometer analysis and quantified events were reported. Data are percentage of GFP^+^ gametocytes (from line 164/GFP) acquired with flow cytometer FACSAria (before sorting) and with imaging cytometer Imagestream 100 (after sorting).

## Discussion

One of the major obstacles in antimalarial drug development is that the parasites develop resistance faster than drug development can follow. As gametocytes mediate the transition from human to insect host, they are an attractive target of chemotherapeutic intervention. However, gametocyte biology remains poorly understood. This is due, in part, to the lack of tools for gametocyte purification and separation. Gametocytes are rare in the infected mammalian host compared to the high asexual stage parasite numbers and can also only be generated in limited numbers *in vitro*, especially when isolation of a specific and synchronous gametocyte stages is desired. Another technical limitation in *P. falciparum* gametocyte research is the nearly inevitable contamination of gametocyte cultures with asexual parasites, which impedes purification of parasites at highly synchronous, young transmission stages of development (before Stage II).

One major problem in the quantification and sorting of parasites samples using a single fluorescent marker with low signal is that the level of AF can produce low signal to noise ratio. From the point of view of minimizing AF, the use of red proteins is preferable, since AF diminishes dramatically with increased excitation wavelength. However, GFP is superior for microscopic observations and single-cell studies compared to many red proteins because of its relatively high brightness and photostability [21,22]. Thus, GFP is most often used for cell sorting due to the convenience of further observations despite rather high AF of cells in this part of emission spectrum. Here, using transgenic *P. falciparum* lines expressing GFP, an optimized protocol for the detection and isolation of parasites at high yield and purity was established.

AF has a very broad spectrum because it is induced by multiple sources in animal cells, including tryptophan, flavins, NAD-H, NAD(P)-H, lipofuscins, porphyrins, etc. [23-25]. The exact pattern and level of AF is related to the cell type, functional state of the cell, cellular morphological features, and fixation status of the specimen [26,27]. Because of its broad spectrum, AF can be distinguished from specific fluorescence emitted by fluorochromes and/or FPs. Most of the AF in the green spectral region is attributed to flavins and flavoproteins [15,16,28], and the AF in the red spectrum is due to porphyrins [29]. Red blood cells also have significant AF after 620 nm [10], which complicates the detection of the far-red fluorescent proteins like mCherry. As shown in Figure 1, good results with transgenic parasites expressing tdT, which has an emission peak in a region where AF from red blood cells is low, were obtained.

However, because of the relatively low photostability of tdT, GFP is much more commonly used in transgenic lines in *Plasmodium* studies. Therefore, it is important to have an optimized protocol for its detection by flow cytometry. Until now, 530/30 or 530/40 filters were primarily used for the acquisition of GFP-expressing cells in the majority of malaria-related publications (Additional file 4). According to the spectral data, these filters are not optimal for the acquisition of GFP-expressing parasites, since signal acquisition with such filters leads to the loss of a large part of GFP signal and, consequently, GFP-expressing cells. However, standard filters supplied with instruments for the FL1 channel on the majority of cytometers (530/30) continue to be used for sorting and analysis of other GFP-expressing cells and bacteria [30], even for quantitative analysis of low numbers of GFP^+^-cells (concentrations <0.1%, i.e. rare cell subpopulations) [31-33]. In the case of the FACSCalibur instrument, there is no other choice of filters for FL1 detection, and the investigator ends up sacrificing detection yield by using the built-in optical path. Modern cytometers, in the other hand, have interchangeable filters (Additional file 5), allowing users to optimize detection of events in individual channels. For example, optimization of the BP filter for FL1 to detect GFP signal is essential, as its emission spectrum is significantly different from the FITC spectrum.

To improve GFP detection and sorting yield for *P. falciparum* research, a strategy was employed to increase the ratio between specific GFP signal and AF signal by using a narrow BP filter that permits the maximum intensity of GFP emission. Since the emission peak of GFP is rather close to the wavelength of a blue laser (509 nm vs 488 nm), only filters with a relatively high steepness of transmission curve could be used. Filters with optical density (OD) at 488 nm of more than six OD units combined with dichroic mirror with >50% transmission at wavelengths longer than 502 nm were selected. Using narrow BP filters only slightly affects the mean fluorescence intensity (MFI) [34], but it significantly reduces the AF background. An important factor in rare cell sorting is the difference between the MFIs of positive and negative subpopulations (i.e., between the background and the true signal) as the presence of highly autofluorescent cells complicates discrimination of positive events from the autofluorescent background [35]. Besides, in order to separate highly autofluorescent cells from the GFP population, one-dimensional analysis by histogram analysis is insufficient. Instead, GFP vs. AF must be plotted on a two-dimensional dot plot using a different channel from the same laser for the y axis (i.e. FL2 channel-Figure 1).

Through use of the above approach it was found that careful choice of BP filters for acquisition of red blood cells infected with GFP-expressing parasites is critical and made it possible to increase yield by 50 to 150% thereby still maintaining 95% purity, and markedly cut sorting time. Optimization of sorting conditions resulted in an insignificant difference between the yield of tdT-expressing, where AF is only a minor issue, and GFP-expressing cells.

The optimized sorting protocol was used with the gametocyte-producing transgenic parasite line (164/GFP), as well as sorting the schizont-stage and the ring-stage of transgenic malaria parasites. The data described here were generated using transgenic parasite lines in which the GFP constructs were maintained episomally. As mentioned above, GFP has become the marker of choice for transgenic studies, since it is readily detected with high specificity and sensitivity by flow cytometry and microscopy. However, there are certain limitations in using GFP as a marker when analysis is performed on fixed specimens: GFP signal may fade over time after fixation, as well as during permeabilization [36,37]. In addition, AF is increased after aldehyde fixation [38] making the MFI difference between positive and negative subpopulations very low. GFP fluorescence is also dependent on the conformation of the protein [39,40]. Thus, modifications of the fixation and permeabilization protocol may be necessary to ensure good GFP signal [41].

FACS sorting of GFP-transfected malaria parasites can be clearly described as “rare cell population” sorting, since transfection efficiencies for parasites are low (0.1-1.0%) [8]. Rare-event cell sorting makes it necessary to sort millions of cells in order to obtain the desired amount of selected cells. Its efficiency depends mainly on the following factors: 1) the signal-to-noise ratio of the population of interest compared to negative population; 2) the expression (%) of the population of interest; and 3) the ratio between cells and debris particles [42,43]. The frequency of false-positive events (noise or impurity) defines the lower limit for detection that is calculated as the upper 99th percentile of negative control.

In conclusion, although there are several limitations associated with the use of GFP in the study of malaria parasite biology, the methodology described in this manuscript overcomes several of these hurdles, and should be immediately applicable for laboratories that have developed GFP-expressing transgenic parasite lines. Maximizing the performance of optical filters has tremendous potential for life science research and cell sorting, in particular rare cell sorting, and allows sorting yield to be markedly increased at a relatively trivial cost compared to the price of the cytometer itself.

## Abbreviations

AF: Autofluorescence; BP: Bandpass; BrdU: Bromodeoxyuridine; BSA: Bovine serum albumine; GFP: Green fluorescent protein; FL: Fluorescent channel; FP: Fluorescent protein; MFI: Mean fluorescence intensity; NAD: Nicotinamide adenine dinucleotide; OD: Optical density; PBS: Phosphate buffered solution; PCR: Polymerase-chain reaction; PFA: Paraformaldehyde; PMT: Photomultiplier; RBC: Red blood cell; tdT: Tandem dimer Tomato fluorescent protein; WT: Wild type.

## Competing interests

PP is employed by Semrock Inc. Other authors do not have any competing interests.

## Authors’ contributions

The work was carried out in collaboration between all authors. IAV and KB designed experiments, carried out the laboratory experiments, analysed the data, interpreted the results and wrote the draft. KK, PP and EE co-designed and performed experiments and contributed in data interpretation and report writing. MM and MD supervised KB and EE, coordinated the research, and commented on the manuscript. NSB designed and coordinated research, analysed the data, wrote the manuscript, and provided funding. All authors have contributed to, seen, and approved the final manuscript.

## Supplementary Material

Additional file 1 Spectral characteristics of the optical filters used in the study (A) and the transmission spectra of three different optical filters used for the detection of GFP-positive events superimposing the GFP emission spectrum (B). Click here for file

Additional file 2 **Relative yield (%) of GFP**^**+**^** gametocytes (line 164/GFP) collected with a FACSAria cytometer, equipped with dichroic filter 466LP vs a FACSAria cytometer equipped with 502LP dichroic and different bandpass filters.** Click here for file

Additional file 3 **Representative image gallery of sorted red blood cells infected with GFP-expressing parasites (line 164/GFP).** The parasites were sorted using a 517/20 filter (dichroic 502LP) and images were acquired with Imagestream 100 imaging cytometer. Left channel: bright field, middle channel: fluorescent channel 3 (GFP-channel), right channel: merged bright field and green channel. Click here for file

Additional file 4 Cytometer and optical filter information available from publications on malaria research. Click here for file

Additional file 5 List of optical filters interchangeability in some cytometers. Click here for file
