# Supplementary figures and images for: Optimization of flow cytometric detection and cell sorting of transgenic Plasmodium parasites using interchangeable optical filters
Source: Malar J. 2012 Sep 5;11:312. doi: 10.1186/1475-2875-11-312 (PMC3544587; doi:10.1186/1475-2875-11-312)

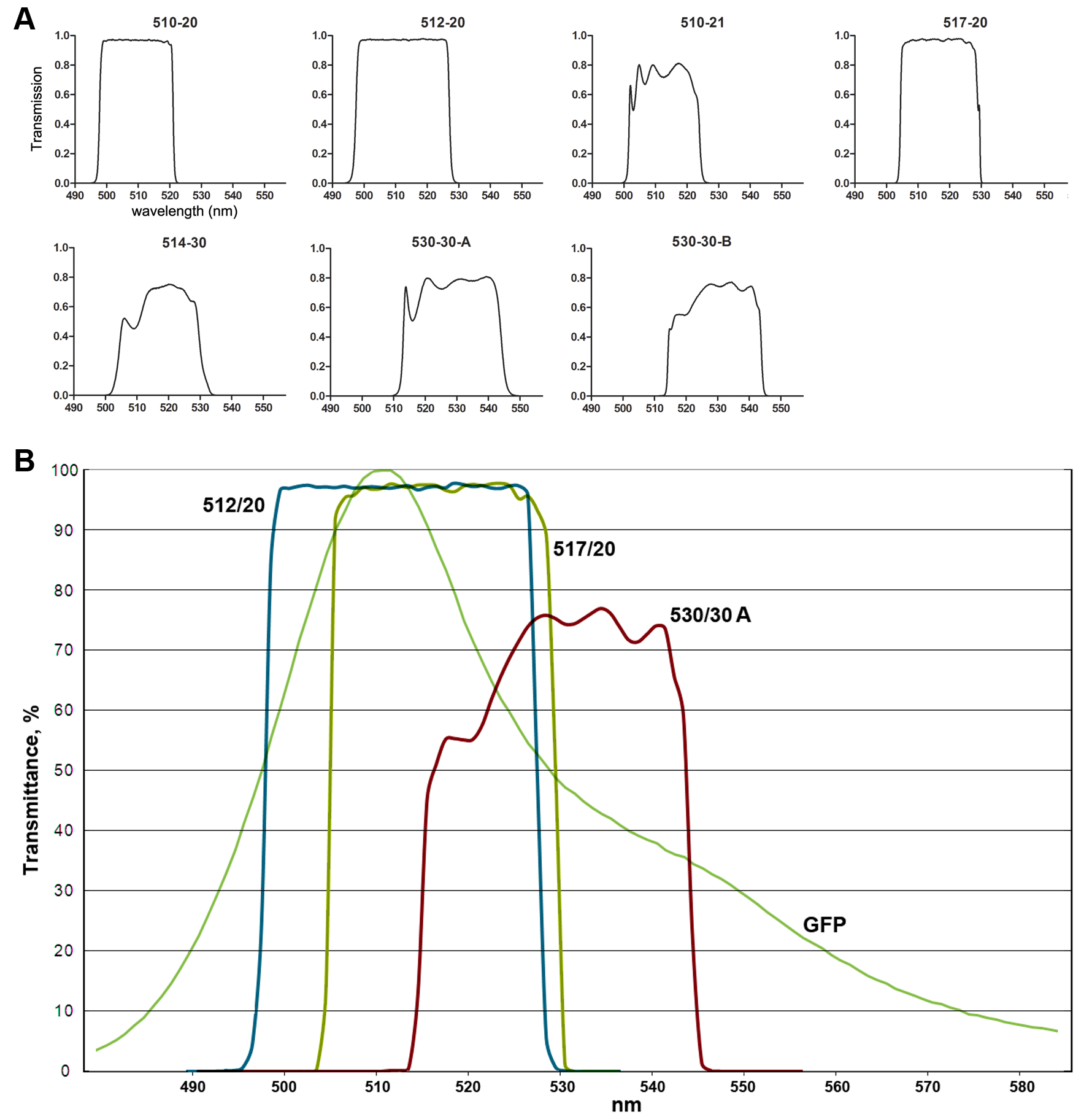

Supplement: Additional file 1 — Spectral characteristics of the optical filters used in the study (A) and the transmission spectra of three different optical filters used for the detection of GFP-positive events superimposing the GFP emission spectrum (B). [file 1475-2875-11-312-S1.tiff]

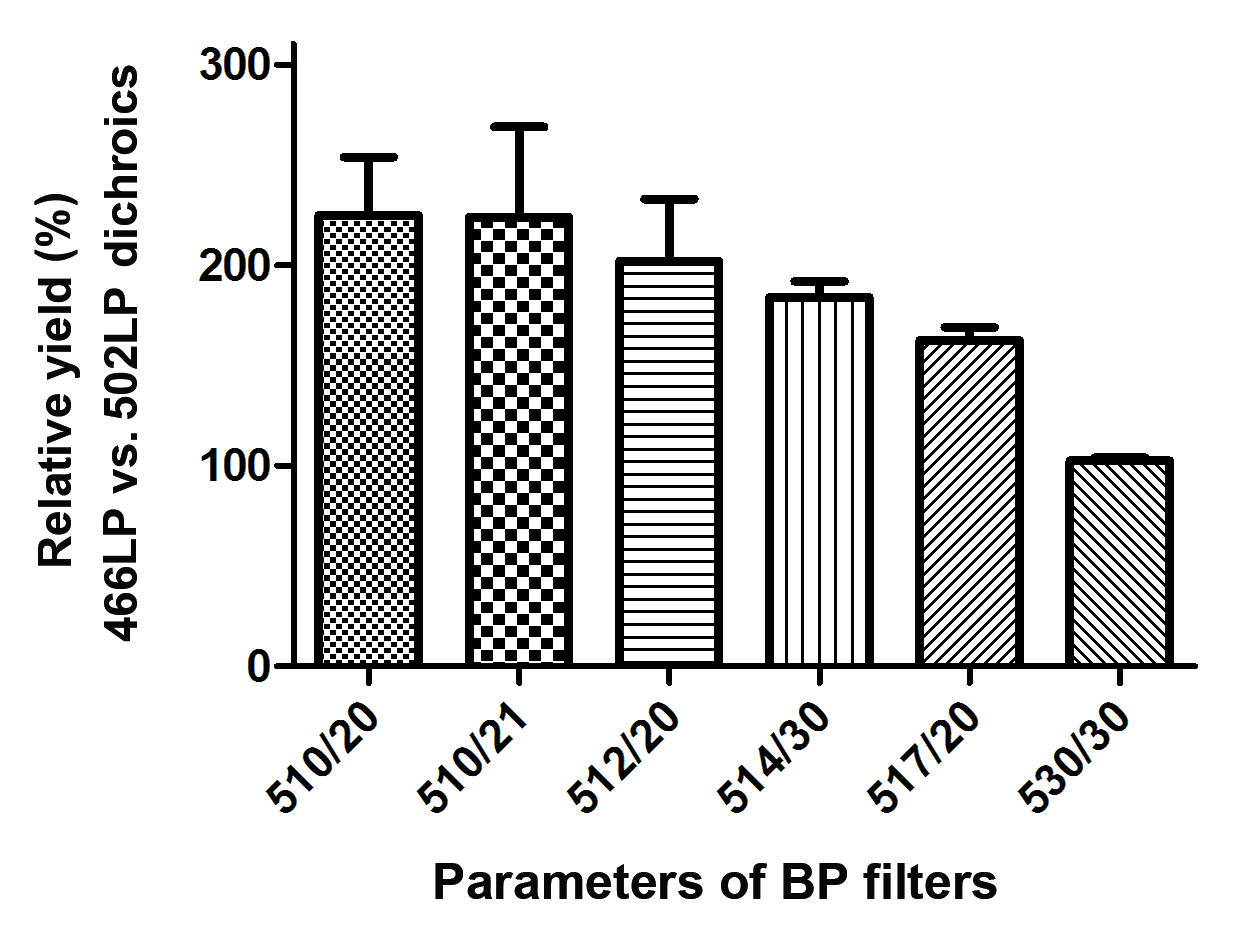

Supplement: Additional file 2 — Relative yield (%) of GFP+ gametocytes (line 164/GFP) collected with a FACSAria cytometer, equipped with dichroic filter 466LP vs a FACSAria cytometer equipped with 502LP dichroic and different bandpass filters. [file 1475-2875-11-312-S2.tiff]

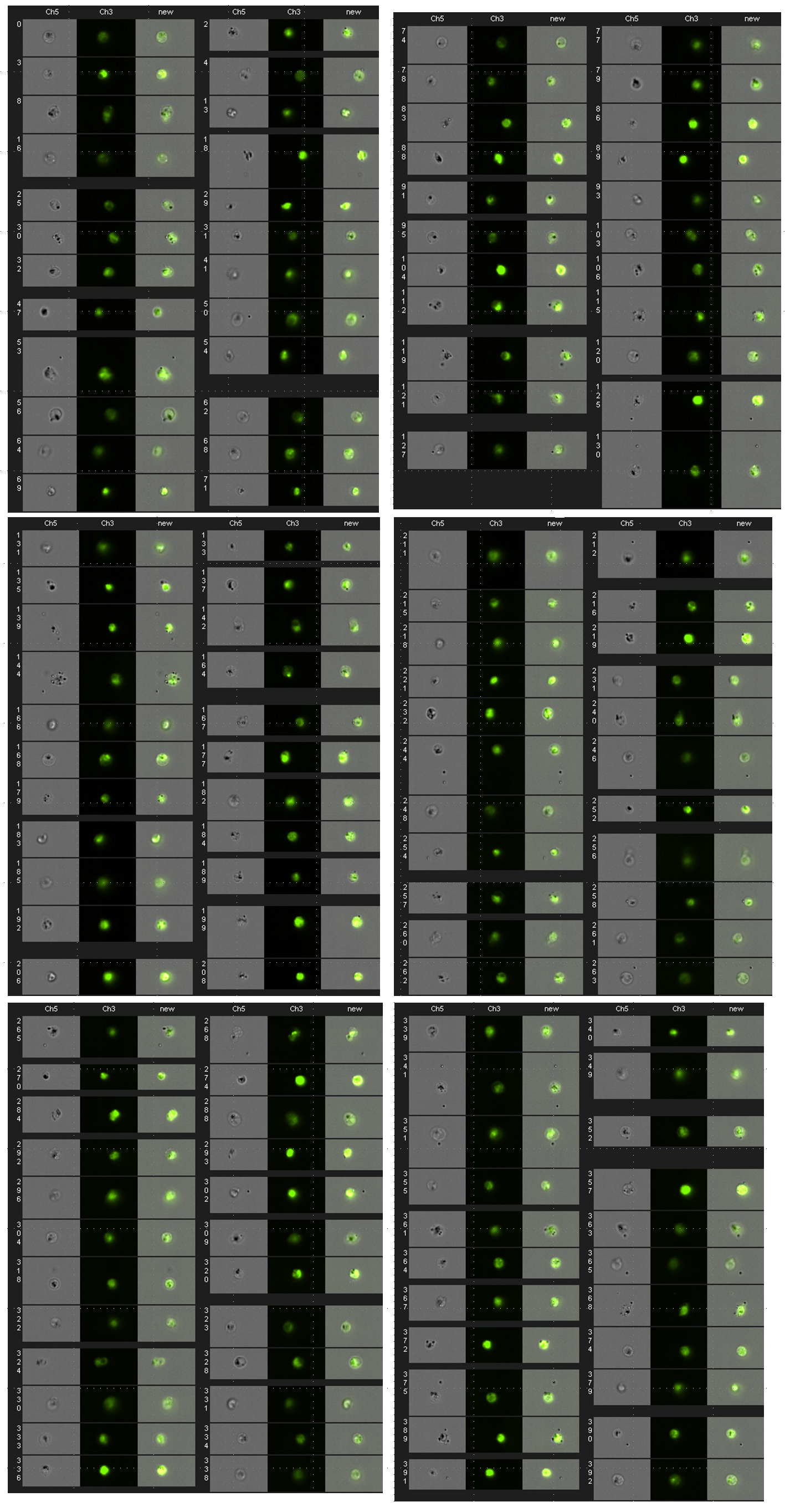

Supplement: Additional file 3 — Representative image gallery of sorted red blood cells infected with GFP-expressing parasites (line 164/GFP). The parasites were sorted using a 517/20 filter (dichroic 502LP) and images were acquired with Imagestream 100 imaging cytometer. Left channel: bright field, middle channel: fluorescent channel 3 (GFP-channel), right channel: merged bright field and green channel. [file 1475-2875-11-312-S3.tiff]
