# Supplementary material for: Optimization of flow cytometric detection and cell sorting of transgenic Plasmodium parasites using interchangeable optical filters
Source: Malar J. 2012 Sep 5;11:312. doi: 10.1186/1475-2875-11-312 (PMC3544587; doi:10.1186/1475-2875-11-312)
Supplement: Additional file 4 — Cytometer and optical filter information available from publications on malaria research. [file 1475-2875-11-312-S4.doc]

**Additional File 4.** Cytometer and optical filter information available

from publications on malaria research

| **Reference** | **Cytometer/Optical filter, if available** | **Plasmodium strain** |
| --- | --- | --- |
| ^1^Natarajan et al., 2001 | *FACSScan (BD Biosciences) | *P. berghei*, PbFluspo strain |
| ^2^Franke-Fayard et al, 2004 | FACSScan, **530/30**  FACSVantage, not specified | *P. berghei, PbGFP_CON_  line* |
| ^3^Tarun et al., 2006 | Influx, **530/40** | *P. yoelii*, PbGFP and PyGFP lines |
| ^4^Janse et al., 2006 | FACSVantage-DiVa, and/or FACSAria, **530/30** | *P. berghei*, 354cl4 (PbGFP-LUC_SCH_)3 |
| ^5^Nkrumah LJ et al., 2006 | LSRII (BD Biosciences), **530/30** | *P. falciparum*, Dd2/GFP and Dd2attB/GFP lines |
| ^6^Ono T et al., 2007 | *FACSCalibur | *P. yoelii yoelii* 17XNL GFP^+^ line |
| ^7^Prudencio et al., 2008 | *FACSCalibur | *P. berghei*, line 259 cL2, PbGFP and RASPbGFP |
| ^8^Talman et al., 2010 | *FACSCalibur | *P. falciparum*, 3D7HT-GFP^+^ strain |
| ^9^Wilson et al., 2010 | *FACSCalibur | *P. falciparum*, D10-PcPHG and PfPHG lines |
| ^10^Boyle et al., 2010 | *FACSCalibur; info from other publications of this group | *P. falciparum*, D10-PfPHG |
| ^11^Shiratsuchi T et al., 2010 | *FACSCalibur | *P. falciparum* and *P. yoelii*, different GFP^+^ lines |
| ^12^Talman et al, 2010 | *FACSCalibur | 3D7HT-GFP *P. falciparum* |
| ^13,14^Miao et al., 2010, 2011 | Influx (BD Biosciences), **530/40** | GFP*^+^ P. falciparum* |
| ^15^Buchholz et al., 2011 | Lab Quanta (Beckman Coulter), **525** (width of bandpass filter is not specified) | *P. falciparum*, 3D7 strain, P2G12 clone |
| ^16^Gerena et al., 2011 | FACSCalibur (BD Biosciences); **530/30** | *Plasmodium berghei* ANKA (clone 15cy1), ANKA GFP^+^ (507cl1, 20) |

*****FACSCalibur and FACScan (BD Biosciences, San Jose, CA) - cytometers with non-

interchangeable optical filters and with standard 530/30 filter in FL1 (fluorescent channel

or green channel position).

Influx, FACSAria, LSRII (BD Biosciences) and Lab Quanta (Beckman Coulter) -

cytometers with interchangeable filters.

**References from Additional Table 4:**

1.Natarajan R, Thathy V, Mota MM, Hafalla JCR, Menard R, Vernick KD: **Fluorescent**

***Plasmodium berghei* sporozoites and pre-erythrocytic stages: a new tool to study**

**mosquito and mammalian host interactions with malaria parasites**. *Cell Microb*

2001, **3**: 371-379.

2.Franke-Fayard B, Trueman H, Ramesar J, Mendoza J, der Keur et al: **A *Plasmodium***

***berghei* reference line that constitutively expresses GFP at a high level throughout**

**the complete life cycle.** *Mol Biochem Parasitology* 2004, **137**: 23-33.

3. Tarun AS, Baer K, Dumpit RF, Gray S, Lejarcegui N et al: **Quantitative isolation and**

**in vivo imaging of malaria parasites liver stages**. *Inter J Parasit* 2006, **36**: 1283-1293.

4. Janse CJ, Franke-Fayard B, Waters AP: **Selection by flow-sorting of genetically**

**transformed, GFP-expressing blood stages of the rodent malaria parasite,**

***Plasmodium berghei*.** *Nature Protocols* 2006, **1**: 620 - 623.

5. Nkrumach LJ, Muhle RA, Moura PA, Ghosh P, Hatfull GF, Jacobs WR, Fidock DA:

**Efficient site-specific integration in *Plasmodium falciparum* chromosomes mediated**

**by mycobacteriophage Bxb1 integrase.** *Nat Methods* 2006, **3**: 615-621.

6. Ono T, Tadakuma T, Rodriguez A: ***Plasmodium yoelii yoelii* 17XNL constitutively**

**expressing GFP throughput the life cycle.** *Exp Parasitol* 2007, **115:** 310-313.

7. Prudencio M, Rodrigues CD, Ataide R, Mota MM: **Dissecting in vitro host cell**

**infection by *Plasmodium* sporozoites using flow cytometry.** *Cell Microb* 2008, **10**:

218-224.

8. Talman AM, Blagborough AM, Sinden RE: **A *Plasmodium falciparum* strain**

**expressing GFP throughout the parasite's life-cycle.** *PLoS One* 2010, **5**:e9156.

9. Wilson DW, Crabb BS, Beeson JG: **Development of fluorescent *Plasmodium***

***falciparum* for in vitro growth inhibition assays.** *Malaria J* 2010, **9**: 152.

10. Boyle M.J., Wilson D.W., Richards J.S. et al. **Isolation of viable *Plasmodium***

***falciparum* merozoites to define erythrocyte invasion events and advance vaccine**

**and drug development.** PNAS 2010, **107**: 14378-14383.

11. Shiratsuchi T, Rai U, Krause A, Worgall S, Tsuji M: **Replacing adenoviral vector**

**HVR1 with a malaria B cell epitope improves immunogenicity and circumvents**

**pre-existing immunity to adenovirus in mice.** *J Clin Invest* 2010, **120:** 3688-3701.

12.Talman AM, Blagborough AM, Sinden RE: **A *Plasmodium falciparum* strain**

**expressing GFP throughout the parasite's life-cycle.** *PLoS One* 2010, **5**:e9156

13. Miao J, Li X, Cui L: **Cloning of *Plasmodium falciparum* by single-cell sorting.** *Exp*

*Parasitol* 2010, **126**: 198-202.

14. Miao J, Cui L: **Rapid isolation of single malaria parasite-infected red cells by cell**

**sorting**. *Nat Protoc* 2011, **6**: 140-146.

15. Buchholz K, Burke TA, Williamson KC, Wiegand RC, Wirth DF, Marti M: **A**

**high-throughput screen targeting malaria transmission stages opens new avenues**

**for drug development**. *J Infect Dis* 2011, **203***:* 1445-1453.

16. Gerena Y, Gonzalez-Pons M, Serrano AE: **Cytofluorometric detection of rodent**

**malaria parasites using red-excited fluorescent dyes**. *Cytometry A* 2011, **79**: 965-972.
