# Supplementary material for: Optimization of flow cytometric detection and cell sorting of transgenic Plasmodium parasites using interchangeable optical filters
Source: Malar J. 2012 Sep 5;11:312. doi: 10.1186/1475-2875-11-312 (PMC3544587; doi:10.1186/1475-2875-11-312)
Supplement: Additional file 5 — List of optical filters interchangeability in some cytometers. [file 1475-2875-11-312-S5.doc]

**Additional File 5.**

List of optical filters interchangeability in some cytometers.

| Instrument | Company | Filter (standard BP filter supplied for FL1 channel of cytometer) |
| --- | --- | --- |
| LSR II | BD Biosciences | 530/30; interchangeable |
| LSR Fortessa | BD Biosciences | 530/30; interchangeable |
| FACSCanto | BD Biosciences | 530/30; interchangeable |
| FACSAria | BD Biosciences | 530/30; interchangeable |
| Influx | BD Biosciences | 530/40; interchangeable |
| FACSVerse | BD Biosciences | 530/30; Pre-assembled filter units with built-in memory chips |
| MoFlo | Beckman Coulter | 529/28; interchangeable |
| Astrios | Beckman Coulter | 530/30; interchangeable |
| Gallios | Beckman Coulter | 525/40; interchangeable |
| CyAn | Beckman Coulter | 530/40; filter sticks are interchangeable |
| FC500 | Beckman Coulter | Bandpass 525 nm; interchangeable |
| Epics XL | Beckman Coulter | 525/30; No filters exchange |
| Cell Lab Quanta SC | Beckman Coulter | Bandpass 525 nm; interchangeable |
| S1000 series | Stratedigm | 530/30; interchangeable |
| S1000EX series | Stratedigm | 530/30; interchangeable |
| SE500 series | Stratedigm | 530/30; interchangeable |
| MACSQuant analyzer | Miltenyi Biotec | 525/50; No filters exchange |
| FACSCalibur | BD Biosciences | 530/30; No filters exchange |
| FACSScan | BD Biosciences | 530/30; No filters exchange |
| Accuri C6 | BD Biosciences | Swappable filter 533/30 nm |
| Sy3200 | iCyt | 525/50, interchangeable filters and PMTs modules |
| ec800 (Eclipse) | iCyt | 525/50; No filters exchange |
| Guava EasyCyte 8HT | Millipore | 525/30; No filters exchange |

- The table is based on technical characteristics available from company and university core facilities websites. Most of instruments can be customized.
- Standard configurations of above-mentioned cytometers optimized for FITC and/or FITC/GFP acquisition
